# Bacterial chondronecrosis with osteomyelitis related *Enterococcus cecorum* isolates are genetically distinct from the commensal population and are more virulent in an embryo mortality model

**DOI:** 10.1186/s13567-023-01146-0

**Published:** 2023-02-23

**Authors:** Yue Huang, Venessa Eeckhaut, Evy Goossens, Geertrui Rasschaert, Johan Van Erum, Geert Roovers, Richard Ducatelle, Gunther Antonissen, Filip Van Immerseel

**Affiliations:** 1 Livestock Gut Health Team (LiGHT), Department of Pathobiology, Pharmacology and Zoological Medicine, Faculty of Veterinary Medicine, Ghent University, Salisburylaan 133, 9820 Merelbeke, Belgium; 2 Flanders Research Institute for Agriculture, Fisheries and Food (ILVO)- Technology and Food Science Unit, 9090 Melle, Belgium; 3 Galluvet, Dwarsstraat 3, 3560 Lummen, Belgium

**Keywords:** Bacterial chondronecrosis with osteomyelitis (BCO), broiler, *Enterococcus cecorum*, chicken

## Abstract

Bacterial chondronecrosis with osteomyelitis (BCO) is a common cause of broiler lameness. Bacteria that are found in BCO lesions are intestinal bacteria that are proposed to have translocated through the intestinal epithelium and have spread systemically. One of the specific bacterial species frequently isolated in BCO cases is *Enterococcus cecorum.* In the current study, caecal isolates were obtained from birds derived from healthy flocks (12 isolates from 6 flocks), while isolates derived from caeca, colon, pericardium, caudal thoracic vertebrae, coxo-femoral joint, knee joint and intertarsal joint (hock) were obtained from broilers derived from BCO outbreaks (111 isolates from 10 flocks). Pulsed field gel electrophoresis was performed to determine similarity. Clonal *E. cecorum* populations were isolated from different bones/joints and pericardium from animals within the same flock, with intestinal strains carrying the same pulsotype, pointing to the intestinal origin of the systemically present bacteria. Isolates from the intestinal tract of birds from healthy flocks clustered away from the BCO strains. Isolates from the gut, bones/joints and pericardium of affected animals contained a set of genes that were absent in isolates from the gut of healthy animals, such as genes encoding for enterococcal polysaccharide antigens (*epa* genes), cell wall structural components and nutrient transporters. Isolates derived from the affected birds induced a significant higher mortality in the embryo mortality model as compared to the isolates from the gut of healthy birds, pointing to an increased virulence.

**Supplementary Information:**

The online version contains supplementary material available at 10.1186/s13567-023-01146-0.

## Introduction

The poultry industry is providing an affordable source of high quality protein for the growing human population. To meet this growing demand for animal derived proteins, decades of genetic selection have created broiler chickens that are able to consume huge amounts of feed and convert it very efficiently into meat. The downside, however, is that the birds are more susceptible to metabolic and infectious diseases that are directly linked to the high performance, sometimes grouped under the general heading of production diseases. Considering the fast weight gain and the immaturity of the skeleton, it is not surprising that one of the most important production diseases of the last two decades is bacterial chondronecrosis with osteomyelitis (BCO), often presenting as lameness, and sometimes even inability to walk [1]. This disease is one of the most common causes of lameness in broilers [2, 3], and is characterized by bacterial infection in rapidly growing bones under repeated mechanical stress and typically occurs in tibiae, femora and the caudal thoracic vertebrae. The terminology is often confusing and names such as “kinky back” (incorrectly), spondylitis, spondylolisthesis and femoral head necrosis are given to describe similar or equal syndromes [4]. The term BCO refers to a bacterial infection which differentiates it from non-infectious causes of lameness [2, 3, 5, 6].

The pathogenesis of BCO is not fully understood although it is assumed that bacteria enter the bloodstream and hematogenously spread to the osteochondrotic clefts or to microfractures at the growth plates [2, 6, 7]. When colonizing the growth plates, the bacteria are rather inaccessible to antibiotics and the host immune system, making them able to induce necrosis.

Strikingly, bacteria that are found in BCO lesions are mostly commensal intestinal bacteria that have translocated through the intestinal epithelium and have spread systemically. This points to a clear connection between gastrointestinal health and BCO, supported by the observation that probiotics can aid to prevent BCO [8, 9]. Bacterial genera and species that are isolated from BCO cases are, amongst others, opportunistic bacteria including staphylococci, *Escherichia coli*, and enterococci. While staphylococci are colonizing the upper respiratory tract and skin, *E. coli* and enterococci are abundant intestinal bacteria. One of the specific bacterial species isolated in BCO cases is *Enterococcus cecorum*, a facultative anaerobic, Gram-positive bacterium that is a common inhabitant of the chicken intestinal tract [2, 6, 7]. Isolates of *E. cecorum* derived from osteomyelitis, spondylitis and arthritis cases are mostly clonal, whereas isolates from the ceca of healthy chickens are genetically diverse [10–13]. Spondylitis has been reproduced in male broiler breeders after intravenous as well as oral challenge with *E. cecorum* isolates from spondylitis cases, thus fulfilling Koch’s postulates [14]. However, the exact pathogenesis, the nature and role of these virulence factors in the initiation and development of disease is not understood.

Genomic comparisons between *E. cecorum* strains isolated from lesions of diseased animals with isolates from healthy animals have been performed, and genes that are specifically present in each type have been identified [15, 16]. While this generates useful information, it is not yet clear which genetic elements are contributing to disease. Some genes related to biofilm production and the production of cell surface polysaccharides are hypothesized to be important [15]. Moreover, studies that have been performed use local isolates, so it is not clear whether the same types of strains can be found worldwide. Various studies report that strains isolated from cases differ in the capability to induce mortality after inoculation in embryonated eggs, although this does not seem to be fully conclusive, as some variation has been described [17, 18]. This is speculated to be due to the use of non-pathogenic strains in the group of presumed pathogenic strains, because of isolation of commensals in diseased animals, but also the embryo mortality assays might not be the best indicator of strain abilities to induce BCO [19, 20]. A main problem in the literature until now is that the embryo mortality assays do not describe the genetic background of the strains, but rather use the isolation source to compare the virulence potential.

Therefore, the aim of our study was to determine whether similar genetic differences, as described for US strains, exist between Belgian *E. cecorum* isolates from BCO cases, as compared to strains from healthy broilers, using PCR detection of a subset of hypothesized virulence genes. In addition, we provide a non-causative link between the embryo mortality induced by pathogenic and non-pathogenic strains, with the presence of the virulence genes.

## Materials and methods

### Bacterial isolation and culture

Samples were collected from 16 different broiler farms (10 BCO outbreak farms and 6 farms without disease) in Belgium during 2019–2020. Samples consisted of swabs of bone and joint lesions (caudal thoracic vertebrae, coxo-femoral joint, knee joint, hock joint), pericardium, caecal and colon (from the ileo-caecal junction to the cloaca) content from chickens suspected of *E. cecorum* infection based on clinical history and necropsy findings. Swabs from caeca of clinically healthy birds, originating from 6 different farms with no clinical signs or necropsy findings associated with *E. cecorum*, were also collected. Swabs were inoculated onto Columbia agar with colistin (10 mg/L), nalidixic acid (10 mg/L), and 5% sheep blood (CNA). Plates were incubated overnight at 37 ℃ under a 5% CO_2_ atmosphere. For each plate one to three individual small, grey, non-haemolytic or slightly α-haemolytic colonies were purified. Isolates were identified by matrix-assisted laser desorption/ionization-time of flight mass spectrometry (MALDI-TOF) [21–23]. All isolates were stored at −80 °C in medium containing 7.5% Glucose, 50 mL BHI, 150 ml horse serum.

### Pulsed-field gel electrophoresis

All isolates (*n* = 123) were clustered by pulsed-field gel electrophoresis (PFGE) according to the protocol described by Luey et al. [24] and Borst et al. [11]. Briefly, collected *E. cecorum* colonies from an overnight blood plate were resuspended in TE buffer and adjusted to an OD_600_ of 1.5. Mutanolysin (0.4 U/μL; Sigma) and lysozyme (0.94 mg/mL; Sigma) were added before incubation at 37 °C for 10 min. Proteinase K (0.33 mg/mL; Merck Life Science) was added and followed by addition of an equal volume of melted 1.2% SeaKem® Gold agarose (Lonza) before loading into a plug mold. After solidification at room temperature, the agarose plugs were submerged in ES buffer with proteinase K (0.5 mg/mL) and incubated at 55 °C for 1 h with consistent vigorous shaking. The plugs were then washed twice in sterile Ultrapure water at 50 °C for 10 min, followed by 4 times washing with TE buffer, where after the plugs were kept at 4 °C in TE buffer. The plugs were sliced and equilibrated in Tango restriction buffer (Thermo Fisher Scientific) at room temperature for 10 min. The slices were then digested in fresh restriction buffer with the enzyme *Sma* I (40 U; Thermo Fisher Scientific) at 30 °C for 2 h. Electrophoresis was carried out at 6 V for 21 h with a ramped pulse time of 5–30 s in 1% SeaKem® Gold agarose with 0.5 × Tris–borate–EDTA (TBE) buffer at 14 °C. *Salmonella enterica* serotype Braenderup H9812, digested with *Xba* I (100 U; Thermo Fisher Scientific) was used as standard. The UV-illuminated image of the resulting gels was digitally recorded after ethidium bromide staining and Ultrapure water de-staining. Analysis of the digitalized images was assisted by the software BioNumerics (version 7; Applied Maths, Sint-Martens Latem, Belgium). Similarity was calculated by using Dice with a 1.0% position tolerance and 1.0% optimization, and the Ward clustering method. Isolates were considered to belong to the same pulsotype only if the fingerprints exhibited at least 95% similarity [25]. Indistinguishable PFGE types were confirmed visually.

### PCR amplification

PCR was performed to determine the presence of 18 putative virulence factors related with *E. cecorum*, according to Borst et al. [15]. Forty-four *E. cecorum* strains from different pulsotypes according to the PFGE result were cultured on blood agar plates overnight. DNA was extracted by adding single colonies into 20 μL lysis buffer (10% SDS, 1 M NaOH), addition of 180 μL HPLC water and 5 min of heating at 95 °C. Then the DNA samples were centrifuged at 16 000 *g* for 5 min. The PCR reaction mix contained 5 μL Polymerase Taq platinum (BioMix™ Meridian Bioscience Inc., USA), 0.25 μL of each primer (20 μM), 1 μL DNA and HPLC water up to 10 μL. PCR amplification was carried out as follows: 95 °C for 5 min; 35 cycles of 95 °C for 30 s, 55 °C for 30 s, 72 °C for 1 min; 72 °C for 10 min. PCR products were electrophoresed on a 1.5% agarose gel. Table 1 shows the targeted genes and the primer sequence. A heatmap of the PCR results was created using the pHeatmap package in R (version 4.2.0).

**Table 1:** **Primer sequences used for PCR detection of potential** ***E. cecorum*** **virulence genes **[15].

Gene	Primers (5´-3´)	Amplicon(bp)	Gene function
ECS3_0190	F-TTGGCAGGAATTATCGATGTGATG	336	hypothetical protein
	R-CTAGTTCCTTTCTTCAACCTCGTC		
ECS3_0193	F-ATGGAATTAAAGAAATATGCCAAAAGAATCC	222	hypothetical protein
	R-TTATTTCCATTGATATTCTGGATAACGATTT		
ECS3_0196	F- ATGGAAAGAATAGATTTAACAAATAAGAAA	1083	dTDP-glucose 4,6-dehydratase (EC 4.2.1.46)
	R-CTATTTATAAAACTCTTTATACCATTCTGCAA		
ECS3_0199	F- GTGCTAATAAAAATAGATGATCCAGGACC	543	Undecaprenyl-phosphate galactosephosphotransferase (EC 2.7.8.6) (wchA)
	R- TCAACTCTGCTTGCCCTCCTTTTTATTTA		
ECS3_0200	F-TTGAAACGAATTTTAATAACTGGATTGAATAG	873	UDP-glucose 4-epimerase (EC 5.1.3.2)
	R-TCAAATTTCTGTCCTCTCAATTGATTCA		
ECS3_0201	F- TTGAAAAAGAAAGTCATGTTTTTAGTAAATCA	1095	Gylosyl transferase, cap 1E-like
	R-TTAAAAATTAATTTCAATCTCTACACAATATTTC TTTAC		
ECS3_0202	F-ATGAATAAGTATGTAAGAGCAACATGT	564	Maltose O-acetyltransferase (EC 2.3.1.79)
	R-TTATTTAATAAACTGAATATTTCTAACACTTTTTCC		
ECS3_0203	F-GTGGATAAGGAAGTAATAGTATTTATAGGA	1188	lipopolysaccharide biosynthesis RfbU-related protein
	R- TCACCTCATTTGTACATTTTTTATCAA		
ECS3_0204	F-ATGAGAATTAATATTGCTTATGCTTGTGATG	930	Glycosyl transferase
	R-TTAATTAAAATACAAATATAACTTAACAAATAAAC TTTTGGGT		
ECS3_0205	F-ATGAATATTAGGAAAACAATTCAAAATATGTTC	1422	Membrane protein involved in the export of O-antigen, teichoic acid lipoteichoic acids
	R-TTAATTTGAAATATTTTTCACTTTACAATTT AAAAGTCT		
ECS3_0206	F-ATGAATTTTATTGTTATATTTCTTGTCTCGCTC	1197	hypothetical protein
	R-CTACCTAGAAATTTTTGAACTAAAATAAGCTAGC		
ECS3_0211	F-ATGAATATGTTAGAAATGAAATATGAGCGC	318	FIG00410032: hypothetical protein
	R- TTAAAACCATTGTTCCACACCAGG		
ECS3_0212	F- ATGATTAAAGGGACCTCTGTTGCAAAAAG	561	hypothetical protein
	R-TTACCAAGAGATATTTTTATAATCTTTCTTGAAA		
ECS3_0213	F- ATGGACAAACTTGTACCTGCATTTG	324	hypothetical protein
	R-TTAAAAGAATAATAACTCTGCCAAGTTCCTCT		
ECS3_0662	F-ATGAATGGTATGCTTCATGATTTGAAA	804	Lipoate synthase
	R-TCATTTGCTCGCTCCACCATCGGCTAAT		
ECS3_2294	F-AACGATTTTCAAAGAGAACTTCCTCAG	936	Glycosyltransferase
	R-TACGCAACAATTTAAGAAAAAATGCCTAAG		
ECS3_2299	F-ATGAGTTCGATTGGAGTTTTTAATTCTGTC	1860	*epaP*-like (hypothetical protein)
	R-GGTTGGCATTCAGGTAAAAATTAGA		
ECS3_2316	F-ATGAGTTCGATTGGAGTTTTTAATTCTGTC	1146	FIG00629489: hypothetical protein LPTXG3 domain
	R-ACAAATAGTCAGCTTTTTATTGATAAAATT		

Amplicon sizes and (hypothetical) virulence gene functions are listed.

### Embryo mortality assay

In total 525 fertilized eggs of broilers (Hatchery Vervaeke, Belgium) were used. The eggs were incubated at 37.5 °C and 45% relative humidity. On day 10 of incubation, eggs were inoculated with a selection of 20 *E. cecorum* isolates from lesions of knee joint, caudal thoracic vertebrae (5) and caeca from animals from both healthy flocks (5) and outbreak flocks (10). These 20 strains were selected from different pulsotypes and with different percentages of virulence genes. Strain selection is shown in Additional file 1. Here for, *E. cecorum* strains were cultured in BHI broth for 24 h at 37 °C, where after they were resuspended in sterile phosphate buffer saline (PBS). After that the suspensions were stored at 4 °C for 18 h to 24 h. The number of bacteria was determined by plating ten-fold dilutions on BHI agar plates. *E. cecorum* strains were diluted with sterile PBS to 5 × 10^4^ CFU/mL. The egg fertility was confirmed before inoculation by candling, and infertile eggs and poor quality embryos were removed. The air chamber of all embryos was marked and the shell area was disinfected using Lugol’s iodine solution (Sigma). For each *E. cecorum* strain, 5 eggs of ten days incubation were inoculated with 200 μL/egg of *E. cecorum* suspension (1 × 10^4^ CFU) in the allantoic cavity. Control eggs of ten days incubation were inoculated in the allantoic cavity with 200 μL/egg of sterile PBS. Incubation of inoculated eggs was done for another 7 days, and mortality was evaluated by candling every 24 h. To establish an optimal dose for the embryo mortality assay, we first injected *E. cecorum* strains 3, 89, 102 and 123 in a dilution series (10^2^ CFUs; 10^4^ CFUs; 10^6^ CFUs) in sterile PBS in the allantoic cavity of the eggs. The assay was performed using 5 biological replicates. Statistical analysis was done using a student’s t-test in GraphPad Prism 9.

## Results

### Isolation of *Enterococcus cecorum* strains

In total 76 samples from pericardium, caudal thoracic vertebrae, coxo-femoral joint, knee joint and hock joint were analyzed. Fifty seven percent (13/23) of pericardium samples, sixty four percent (7/11) of coxo-femoral joint samples, ninety two percent (11/12) of caudal thoracic vertebrae samples, seventy nine percent (19/24) of knee joint samples and a hundred percent (6/6) of hock joint samples contained *E. cecorum* isolates. Fifty-eight caecal and 5 colonic samples from outbreak animals were analyzed and 90% (52/58) of the caecal samples and 60% (3/5) of colon samples contained *E. cecorum* isolates. In total 80% (111/139) of BCO samples were *E. cecorum* positive 74% (56/76) of samples from bones/joints and pericardium and 87% (55/63) of samples from caeca and colon). Sixty three percent (12/19) of caecal samples from animals at 6 farms with no clinical problems of BCO contained *E. cecorum* isolates. A description of the characteristics of the 123 *E. cecorum* isolates is shown in Additional file 1.

### Pulsed-field gel electrophoresis

**Figure 1:**
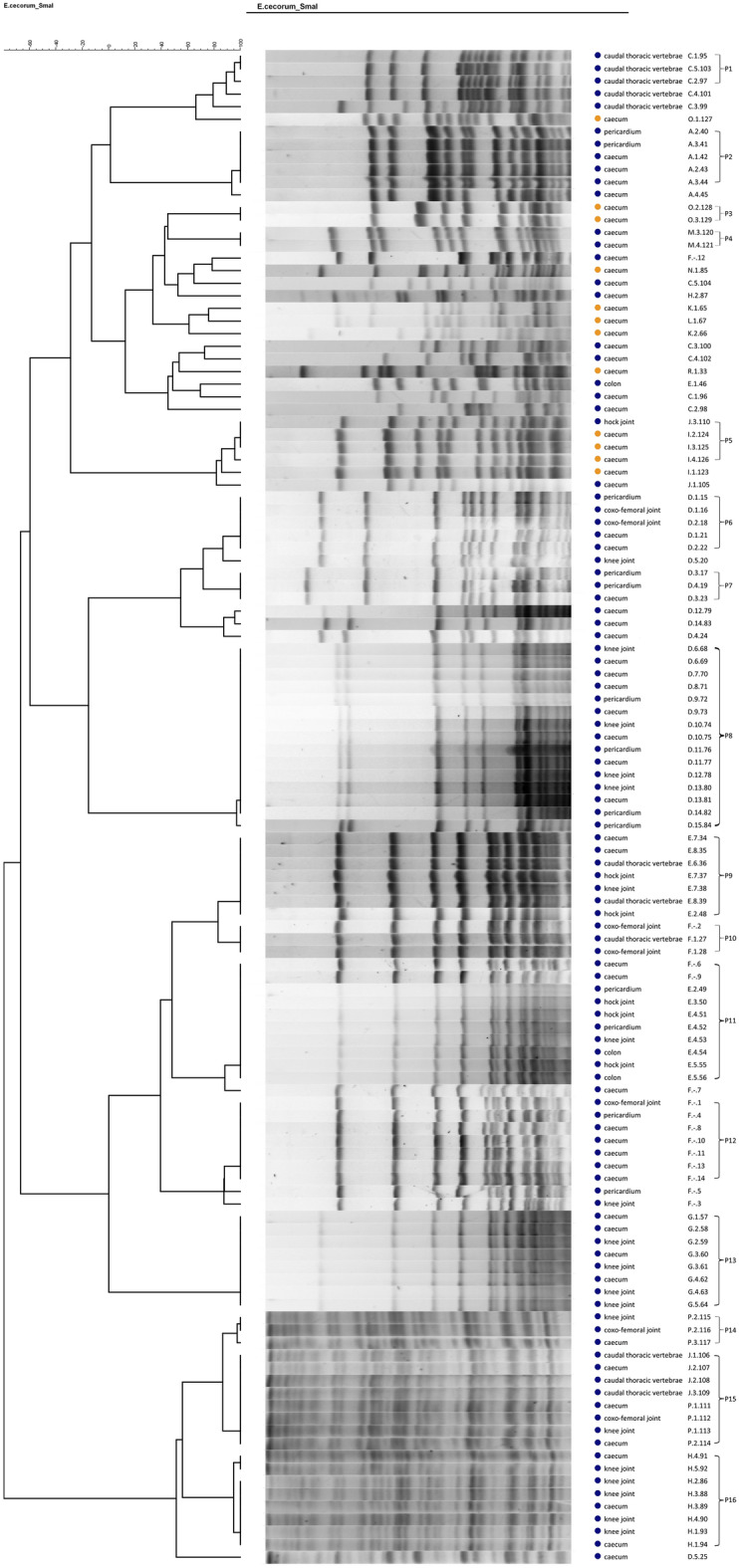
**Pulsed-field gel electrophoresis.** PFGE patterns from *E. cecorum* isolates obtained from sites of isolation from different chickens within different farms. Blue dots: samples from outbreak chickens, orange dots: samples from non-diseased chickens. Each pulsotype is shown with the corresponding *E. cecorum* isolate number (Farm. Chicken number. Isolate number “-” means unknown) and sites of isolation. Analysis shows 16 pulsotype groups (P1-P16), and 27 unique patterns. The dendrogram was generated by cluster analysis using the Ward method and the Dice similarity coefficient.

For all 123 isolates, PFGE was performed to determine similarity (no PFGE profile was available for isolate number 118, 119 and 122; Figure 1). According to the 95% delineation level, the isolates could be grouped in 16 pulsotypes and 27 unique patterns. Clonal *E. cecorum* populations were isolated from different animals and bones/joints and pericardium within the same affected farm, in which intestinal strains carried the same pulsotype, pointing to the intestinal origin of the systemically present bacteria (Figure 1). Isolates from the intestinal tract of broilers from non-affected farms clustered outside the disease-related PFGE clusters.

### PCR-based detection of potential virulence-associated genes

**Figure 2:**
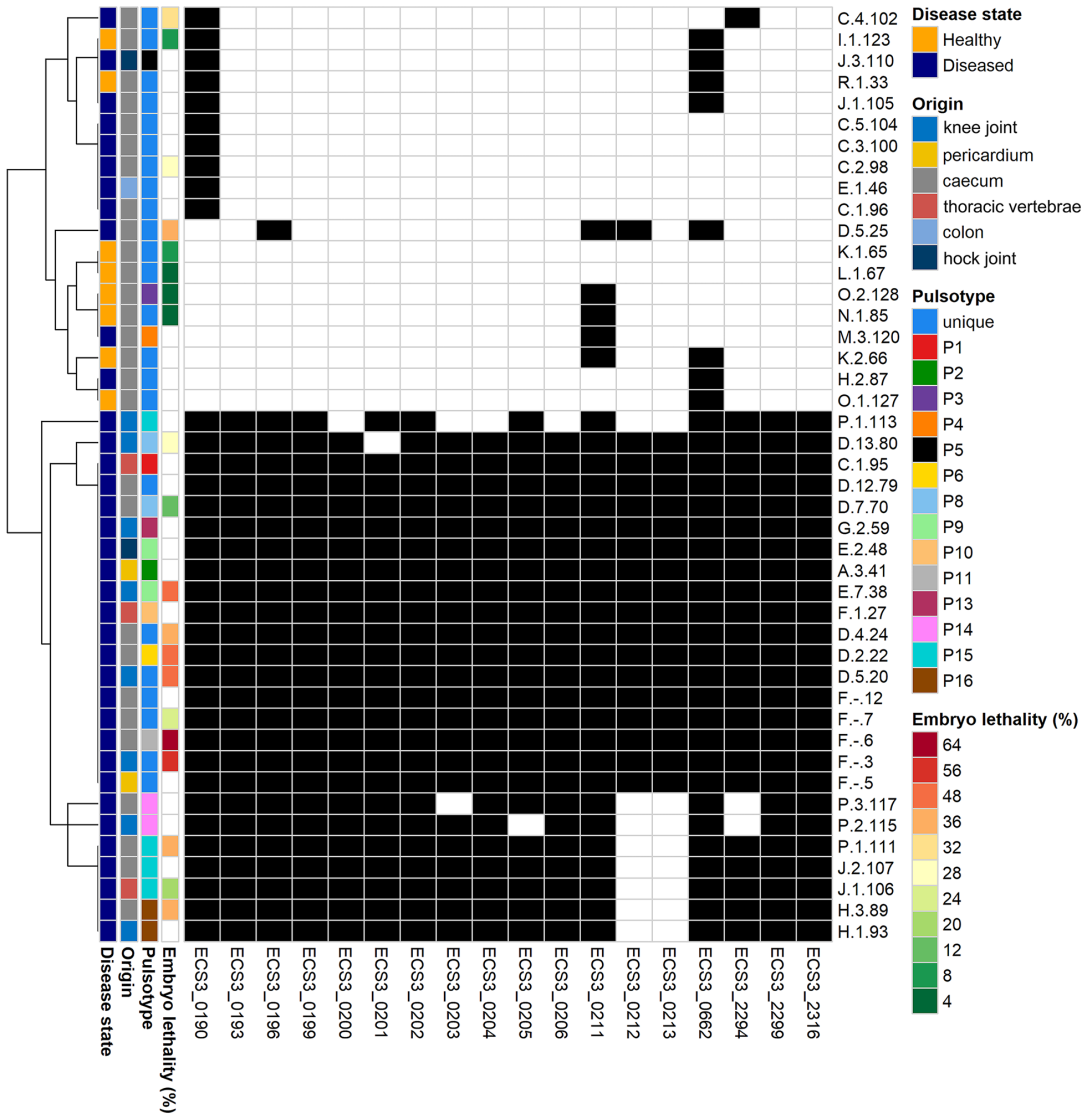
**Heat map of potential virulence factors detected by PCR.** Columns show individual genes (18 potential virulence genes), rows are individual *E. cecorum* isolates. Each tile in the matrix represents the single virulence gene, with presence of a virulence gene as a black tile, and absence as a white tile. Forty-four isolates were tested, out of which F.-.3; F.-.5; D.5.20; F.1.27; E.7.38; A.3.41; E.2.48; G.2.59; D.13.80; H.1.93; C.1.95; J.1.106; J.3.110; P.1.113; P.2.115 isolated from sites of isolation of diseased flocks, F.-.6; F.-.7; F.-.12; D.2.22; D.4.24; D.5.25; E.1.46; D.7.70; D.12.79; H.2.87; H.3.89; C.1.96; C.2.98; C.3.100; C.4.102; C.5.104; J.1.105; J.2.107; P.1.111; P.3.117; M.3.120 isolated from caeca/colon of diseased flocks, R.1.33; K.1.65; K.2.66; L.1.67; N.1.85; I.1.123; O.1.127; O.2.128 isolated from caeca of non-affected flocks.

Based on PFGE results, 36 outbreak strains (3 from thoracic vertebrae, 2 from pericardium, 8 from knee joint, 2 from hock joint, 20 from caeca and 1 from colon) and 8 strains derived from caeca of non-diseased flocks were selected. These were derived from different pulsotypes and unique patterns, and isolated from different birds and bones/joints and pericardium within different farms (Figure 2). Of the 15 *E. cecorum* outbreak strains isolated from bones/joints and pericardium, 14 contained 67–100% of the analyzed potential virulence genes, while 1 contained 11% of these. For the 21 caeca/colon isolates derived from outbreaks, 11 contained 78–100% of the potential virulence genes and 10 contained 6–22% of these. On the other hand, 8 strains isolated from caeca derived from broilers from non-affected flocks, all contained 0–11% of the potential virulence genes (Figure 2). In addition, isolates from the gut, bones/joints and pericardium of affected chickens contained a set of genes that were absent in isolates from the gut of healthy animals, including genes encoding for enterococcal polysaccharide antigen (*epaP*-like gene, ECS3_2299), cell wall structural components and nutrient transporters (Table 1).

### Embryo mortality assay

**Figure 3:**
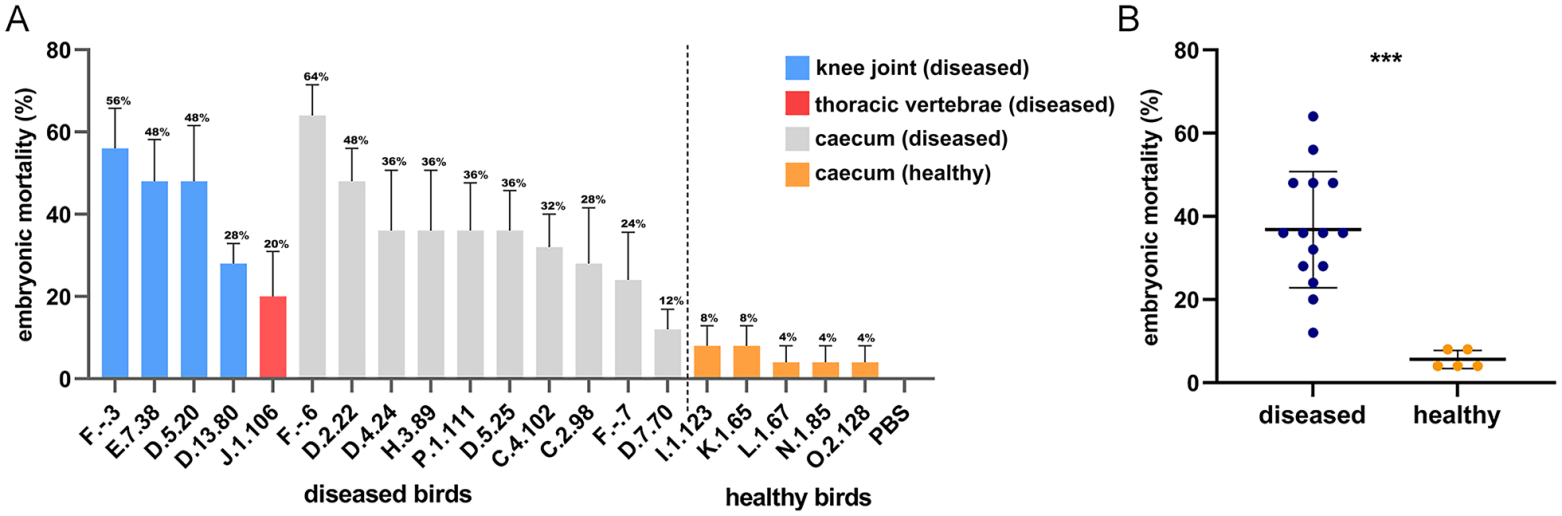
**Embryo mortality percentage for outbreak strains (*n* = 15) and strains isolated from healthy birds (*n*** **= 5).** **A** Used strains were isolates derived from knee joint (blue, *n* = 4), caudal thoracic vertebrae (red, *n* = 1) and caeca (grey, *n* = 10) of diseased birds, and from the caeca of healthy birds (orange, *n* = 5). **B** The average mortality rate of broiler embryos was significantly different between isolates from the diseased as compared to healthy animals (***: *p*-value = 0.0001).

Based on the optimal dose test, in which *E. cecorum* 3, 89 and 102 were outbreak strains and *E. cecorum* 123 was a strain isolated from healthy animals, 10^4^ CFU was chosen as inoculation dose. Injection of *E. cecorum* 3 and *E. cecorum* 89 of 10^4^ CFU and 10^6^ CFU caused an embryo lethality of 80% to 100%. Therefore, we evaluated the embryo mortality caused by 20 isolates at 10^4^ CFU (list of strains used in embryo mortality assay, see Additional file 1). Overall mortality of broiler embryos inoculated with outbreak strains from knee joint/thoracic vertebrae, caeca, and with strains from healthy broilers ranged from 20% to 56%, 12% to 64% and 4% to 8%, respectively. And the average mortality for strains from skeletal lesions was 40% (Figure 3A). The average mortality was 36.8% and 5.6% for outbreak strains and strains isolated from healthy animals (Figure 3B). Isolates derived from the affected birds induced a significant mean higher mortality in the embryo lethality assay as compared to the isolates from the gut of healthy birds (*p*-value = 0.0001). Mortality was mostly seen at day 2 and 3 post-inoculation. We did not find a correlation between the embryo lethality of the strain and the percentage of potential virulence genes encoded by the strain (Figure 2).

## Discussion

Since 2002, outbreaks of joint and bone disease associated with *E. cecorum,* resulting in lameness of broilers, have been increasingly reported in many countries worldwide [6, 26–28]. As an example, a recent study showed that in France, *Enterococcus* represented 0.4% of all reported pathogens in poultry in 2006, growing to 12.9% in 2020 [29]. *Enterococcus cecorum* was mainly associated with locomotor disorders and septicemia [29].

Our data show that clonal *E. cecorum* populations are present in multiple animals in outbreak farms, pointing to the presence of specific pathogenic strains that spread between animals. Using PFGE, we confirmed that pathogenic strains cluster together and into different clusters as compared to commensal strains. This is in agreement with other studies showing farm-specific clonal lineages [30].

Our data show that clonal isolates were derived from the bones/joints and pericardium, but also from the intestinal tract of individual animals, indicating that strains present in the pericardium and bones/joints are gut-derived. In poultry, leakage of the intestinal barrier has been proposed to occur frequently, either induced by pathogens (*Eimeria*, *Clostridium perfringens*, enteric viruses such as adenovirus and reovirus) or by feed-derived molecules (including mycotoxins) [31]. This may allow translocation of intestinal bacteria to the bloodstream. Why specific bacterial pathogens or strains within a bacterial species can translocate, survive the immune defenses of the host, and colonize bones/joints and pericardium is hitherto not clear.

The isolates from outbreaks in Belgium have a genetic profile that is similar to pathogenic clonal bacterial populations in the US, based on PCR-profiling of hypothetic virulence genes. A prior study identified specific genomic regions and genes in US pathogenic strains based on comparative genomics, which may be related to virulence [15]. Our study confirmed these observations, as in our study on average 6.3% of the analyzed putative virulence factors were detected in strains isolated from healthy animals, in contrast to 56%, 75% and 100% in pathogenic strains isolated from caeca/colon, bones/joints and pericardium of diseased animals, respectively. The function of these pathogenicity-associated genes has yet to be investigated, and not all of these genes might have a role in virulence, but could merely be the consequence of the different chromosomal background. Some pathogen-specific genes might have a role though, such as the *epa* gene cluster which is encoding for enterococcal polysaccharide antigens. The *epa* gene cluster plays an important role in the virulence, biofilm formation and resistance to polymorphonuclear leukocyte killing in *E. faecalis* [32–34]. Next steps should be focusing on proving Koch’s Postulates, by removing genes of interest and showing loss of virulence.

The large collection of well-defined *E. cecorum* isolates used in the present study allowed for a clear separation between pathogenic and commensal strains in the embryo lethality assay. Pathogenic strains indeed have virulence attributes that enable them to cause higher embryo mortality as compared to commensal strains (36.8% vs 5.6%). Previous studies also showed higher mortalities caused by outbreak strains, but less obvious differences were observed [18, 20], maybe related to the use of intestinal isolates of healthy animals, that are pathogenic. We indeed used both the origin of the isolates and PCR-based detection of specific genes that are allocated to virulent strains to classify strains as virulent. The embryo mortality model might thus be used to determine the role of specific potential virulence genes in BCO, although it is not entirely clear whether the embryo mortality as such is a reliable indicator of the disease-causing ability in BCO [19, 20].

In conclusion, clonal populations of *E. cecorum* can be isolated from bones/joints and pericardium in BCO outbreaks, and as well in the gut, pointing to the intestinal origin of the strains. Belgian outbreak strains carry the same genetic background as US outbreak strains, and are more virulent in an embryo mortality model as compared to commensal strains. Future steps should be directed to identifying virulence attributes, so that targeted control measures can be developed.

## Supplementary Information

**Additional file 1. Summary of** ***E. cecorum*** **isolates**. Swabs were collected from both diseased (top) or healthy (bottom) birds originating from different farms. In case multiple houses were sampled from the same farm, this is indicated in the column “House”.

## Data Availability

The datasets used and/or analysed during the current study are available from the corresponding author on reasonable request.
